# GoPerio - impact of a personalized video and an automated two-way text-messaging system in oral hygiene motivation: study protocol for a randomized controlled trial

**DOI:** 10.1186/s13063-019-3738-0

**Published:** 2019-12-10

**Authors:** Valentin Garyga, Florian Pochelu, Béatrice Thivichon-Prince, Walid Aouini, Julie Santamaria, France Lambert, Delphine Maucort-Boulch, Francois Gueyffier, Kerstin Gritsch, Brigitte Grosgogeat

**Affiliations:** 1 Faculté d’odontologie, Université de Lyon, Université Lyon 1, Lyon, France; 2 Hospices Civils de Lyon, Service de Consultations et Traitements Dentaires, Lyon, France; 3 Université de Lyon, IGFL UMR 5242, Lyon, France; 4 Université de Monastir, Monastir, Tunisia; 5 CHU de Liège, Liège, Belgium; 6 Hospices Civils de Lyon, Service de Consultations et de Traitements Dentaires, Lyon, France; 7 Université de Liège, Liège, Belgium; 8 Dental Biomaterials Research Unit, Liège, Belgium; 9 Université de Lyon, Université Lyon 1, Lyon, France; 10 Hospices Civils de Lyon, Service de Biostatistique-Bioinformatique, Lyon, France; 11 Laboratoire de Biométrie et Biologie Évolutive, Département Biostatistiques et Modélisation pour la Santé et l’environnement, Université de Lyon, CNRS UMR 5558, Villeurbanne, France; 12 Université de Lyon, LMI UMR CNRS 5615, Lyon, France

**Keywords:** Oral hygiene, Motivational interviewing, Patient education as topic, Patient Compliance, Video, Text messaging, Periodontal diseases, Dental plaque, Telemedicine, eHealth

## Abstract

**Background:**

Oral hygiene is of paramount importance for the preservation of oral health, and for patients affected by periodontal disease establishing an effective oral hygiene routine is the first step of therapy. Several clinical frameworks have been developed to foster behavior change, such as motivational interviewing. However, two obstacles can be identified. First, patients tend to forget the advice they were given during the consultation. Second, it is hard to maintain motivation in the long term, thus leading to relapse. An innovative eHealth solution was designed with the aim to tackle both obstacles and supplement the current clinical standard of care. The primary objective is to compare the full mouth plaque scores of study groups (eHealth plus standard of care versus standard of care only) at 8 weeks of follow up. The main secondary objective is to compare the full mouth bleeding score at 8 weeks of follow up.

**Methods/design:**

The “GoPerio” study is a multicenter, randomized, controlled trial assessing the impact of a novel eHealth concept for oral hygiene motivation (personalized video of oral hygiene routine available for the patient via a cloud server plus interactive text messages) in addition to the current standard of care (motivational interviewing plus tooth scaling and polishing). The minimum sample size required is 86 patients. Participants will be randomized (allocation ratio 1:1): test group (eHealth plus standard of care) versus control group (standard of care only). The primary outcome is oral hygiene as measured by the full mouth (six sites per tooth) plaque control record (PCR) index. The main secondary outcome is gingival inflammation as measured by the full mouth (six sites per tooth) bleeding on probing (BOP) index. Both the primary and the main secondary outcomes are evaluated by blinded and calibrated examiners at 8 weeks of follow up. The other secondary outcomes are patient satisfaction and patient behavior change and motivation.

**Discussion:**

The study will investigate the value of an innovative eHealth approach to strengthen patient motivation for oral hygiene. If proven effective, such an approach would supplement the current clinical standard of care, resulting in improved clinical outcomes with negligible impact on productivity in a dental practice.

**Trial registration:**

ClinicalTrials.gov, NCT03109808. Registered on 12 April 2017.

Sponsor: Hospices Civils de Lyon. BP 2251, 3 quai des Célestins, 69,229 Lyon cedex 02.

Protocol version**:** 1.0 as of 21 September 2016.

## Background

Dental plaque is a bacterial biofilm that is responsible for most periodontal diseases when there is dysbiosis of the oral ecosystem, often due to a lack of oral hygiene [1]. The two main periodontal conditions are gingivitis and periodontitis [2]. In the experimental model of gingivitis, if oral hygiene is reinstated, inflammation is reduced and tissues heal *ad integrum* [3]. If left untreated and compounded by other factors (genetic, environmental, local, etc.), gingivitis evolves to periodontitis in susceptible people [4–6]. In addition to gum inflammation, periodontitis leads to a deeper destructive process targeting the tooth-supporting tissue such as the alveolar bone. Ultimately, this irreversible tissue destruction can lead to the loss of teeth [7]. Other well-documented risk factors for periodontitis include smoking and diabetes mellitus [5, 8–10].

A key aspect of periodontal therapy is the instruction and motivation of patients for a satisfactory level of oral hygiene. Once oral hygiene is established, non-surgical and surgical periodontal care can be initiated. In adults, twice daily toothbrushing and daily interdental care (dental floss or interdental brushes) considerably reduces the amount of dental plaque accumulated on teeth [11–13]. Adequate plaque control reduces the prevalence and the severity of periodontal diseases [14]. It reduces the risk of tooth loss both in healthy patients [15] and in those enrolled in supportive periodontal therapy [16].

Several frameworks have been proposed to improve oral hygiene in periodontal patients, among which one can differentiate those that rely mostly on patient education, those that emphasize the use of technology, and those that combine patient education theories and the use of technology. Behavior change techniques can be used by the practitioner to enhance a patient’s motivation for durable oral hygiene and healthy habits [17]. One such technique is motivational interviewing (MI), a patient-centered communication technique [18]. MI has been successfully implemented in patient motivation in periodontology [19–22] and in smoking cessation advice [23]. Researchers have also proposed several solutions taking advantage of technology. Videos for oral hygiene instruction have not shown any difference when compared to a clinical consultation [24, 25], but the videos in these studie were pre-recorded and generic (i.e. non-personalized). Conversely, having a personalized video filmed by the dentist or the dental hygienist during the consultation, displaying all steps for a correct oral hygiene routine, could be worth exploring as patients may relate more easily to images of their own teeth. Frequent recalls have also been shown to increase long-term adherence in patient motivation for oral hygiene [26, 27], and it is of note that for chronic diseases text-message-based recalls improved adherence to medication in several trials [28–32].

It is possible to combine patient motivation techniques and technological aspects to create innovative eHealth solutions. eHealth is defined as an “emerging field in the intersection of medical informatics, public health and business, referring to health services and information delivered or enhanced through the Internet and related technologies” [33]. The eHealth concept proposed and evaluated in this randomized controlled trial (RCT) features three key aspects: a consultation taking advantage of MI, personalized oral hygiene videos accessible anytime on a smartphone via a cloud portal, and regular and interactive text messages to enhance patient adherence to the oral hygiene routine. To the best of the authors’ knowledge, such a strategy has yet to be evaluated in a RCT.

### Objectives and hypotheses

The aim of this randomized trial is to evaluate an eHealth concept for patient motivation for oral hygiene through the combined use of MI, a personalized oral hygiene video, and motivational interactive text messages.

The primary objective is comparison of the level of oral hygiene as assessed by plaque scores (that range from 0 to 100%) in individuals who benefited from the control versus test patient education strategies at 8 weeks. The main secondary objective is the gingival inflammation as assessed by bleeding on probing (BOP) index (the values of which range from 0 to 100%) of the control and test groups at 8 weeks. Two secondary objectives will be also considered: the satisfaction after 8 weeks and patient motivation for change after 4 and 8 weeks in each treatment arm. Interventions are subsequently further defined.

The underlying hypothesis is that the eHealth concept for patient motivation for oral hygiene would allow better instruction and motivation for oral hygiene because it gives patients constant access to a video summarizing their oral hygiene routine, and patients benefit from regular contact via text messages.

## Methods

### Trial design

The Standard Protocol Items: Recommendations for Interventional Trials (SPIRIT) statement [34] and SPIRIT - Patient-reported Outcomes (SPIRIT-PRO) extension [35] (for patient-reported outcomes) were taken into account in the design of the present clinical trial.

This is a multicenter, randomized controlled trial with two parallel arms. For the primary objective and secondary objectives, only outcome examiners and data analysts are blinded to the allocated intervention (control or test patient education strategy). Patients are randomized at the end of the first consultation so that patients and investigators are not aware of the allocation during the patient education and motivation procedure.

Patients are randomized (allocation ratio 1:1) for the type of patient education strategy (control versus test) with stratification based on center of inclusion, patient gender (male versus female), and tobacco status (currently using tobacco versus currently not using tobacco).

**Fig. 1:**
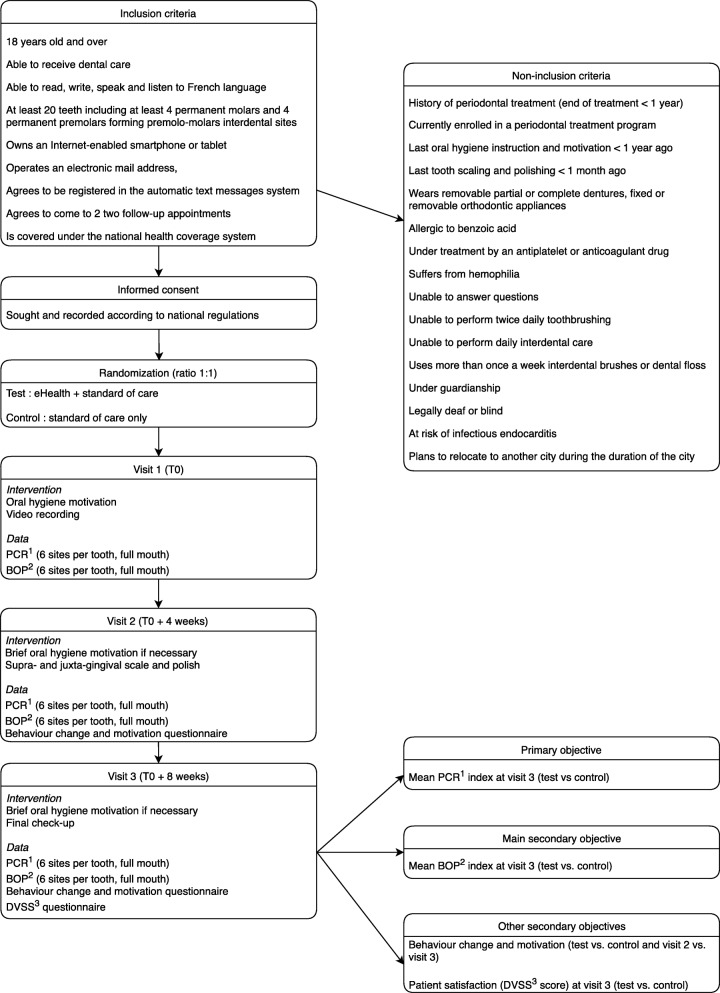
Flow chart. ^1^ PCR = plaque control record. ^2^ BOP = bleeding on probing. ^3^ DVSS = dental visit satisfaction scale

**Fig. 2:**
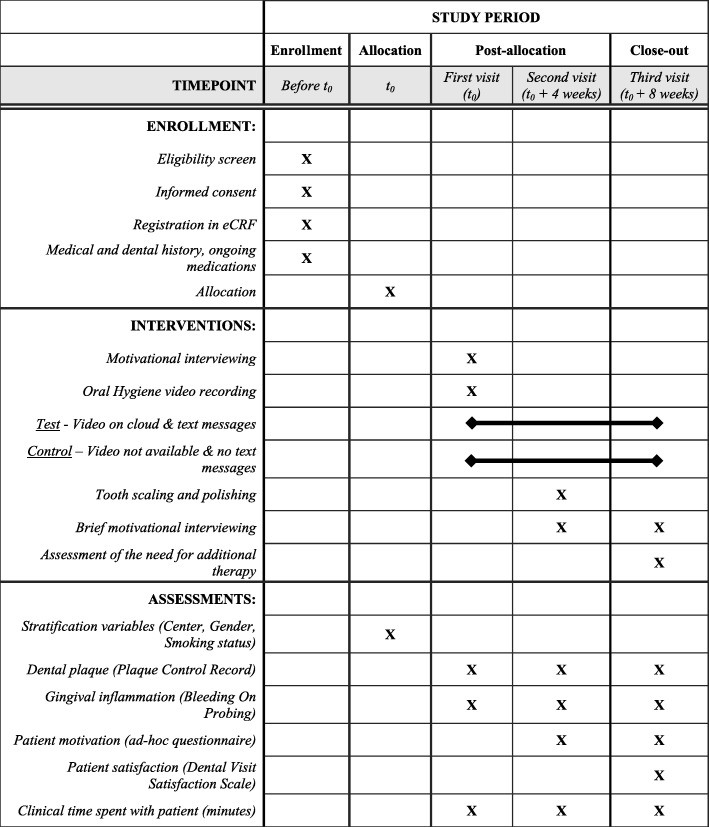
Schedule of enrollment, interventions, and assessments. eCRF, electronic case report form

**Fig. 3:**
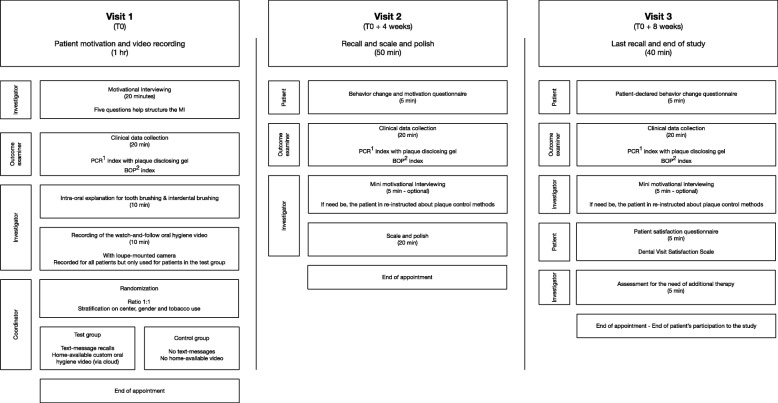
Content of the study visits. ^1^ PCR = plaque control record. ^2^ BOP = bleeding on probing. MI, motivational interviewing

The flow chart of the study is presented in Fig. 1. The schedule of enrollment, intervention, and assessment is reported according to the SPIRIT statement (Fig. 2). The content of each visit is summarized in Fig. 3.

### Setting and participants

This study is carried out in two teaching hospitals, one located in France and the other in Belgium: the dental teaching hospital of the Lyon university hospitals (Lyon, France) and the department of periodontology of the Liège university hospital (Liège, Belgium). Patients will be included from March 2017 until December 2019.

Eligible patients are informed about the study by dentists and dental students working in the hospital. Then, a study investigator presents the study to patients and gives them information letters before inclusion. Ethical approval was obtained in both countries and patient consent is obtained and recorded in accordance with national regulations.

To be included in this trial patients must be 18 years old or over, be able to receive dental care, and be able to read, write, speak, and listen to French language. They must have at least 20 teeth including at least 4 permanent molars and 4 permanent premolars forming premolar-molar interdental sites. They must own an Internet-enabled smartphone or tablet, have an electronic mail address, agree to be registered in the automatic text messages contact system, agree to come to the two follow-up appointments, and be covered under the national health insurance system.

Exclusion criteria are history of periodontal treatment (end of treatment < 1 year), currently enrolled in a periodontal treatment program, last oral hygiene instruction and motivation < 1 year ago, last tooth scaling and polishing < 1 month ago, removable partial or complete dentures, fixed or removable orthodontic appliances, allergy to benzoic acid, under treatment by an antiplatelet or anticoagulant drug, hemophilia, unable to answer questions, unable to perform twice daily toothbrushing, unable to perform daily interdental care, using interdental brushes or dental floss more than once a week, under guardianship, legally deaf or blind, at risk of infectious endocarditis, and planning to relocate to another city during the duration of the study. Non-inclusion criteria have been chosen to reduce loss to follow up or unreliable results.

Medications taken by the patient less than 8 days before entering the study are recorded in the electronic case report form (e-CRF). History of or current renal condition, head and neck cancer, diabetes mellitus, current and past tobacco use, and other current medical conditions must be reported.

No special concomitant care or intervention is prohibited after inclusion in the trial. Patients are specifically instructed to use the material provided for this trial: electric toothbrush (Philips Sonicare, Philips Personal Health France, Suresnes, France); toothpaste (Meridol gum protection, GABA France, Colgate-Palmolive, Bois-Colombes, France); interdental floss (Inava DentoFil Black, Pierre Fabre Oral Care, Castres, France and Meridol expanded dental floss, GABA France, Colgate-Palmolive, Bois-Colombes, France); interdental brushes (Inava Monocompact and Inava Trio Compact, Pierre Fabre Oral Care, Castres, France and Elmex interdental brushes, GABA France, Colgate-Palmolive, Bois-Colombes, France).

### Sample size

A literature review found that behavioral interventions, similar to the patient education strategy explored in this trial, allow up to 15% reduction of dental plaque [21, 36–39]. However, to the best of our knowledge, there is no publication examining the eHealth concept for patient motivation for oral hygiene proposed herein. A conservative estimate of 10% reduction in PCR index was therefore chosen for the test group compared to the control group.

A total of 86 patients enables us to show a 10% difference in the PCR index between the two groups with 80% power, using a bilateral test with an alpha risk of 5%. At 8 weeks, 15% of patients are expected to be lost to follow up (calculated by nQuery Advisor 7.0) [40].

To encourage recruitment, the investigators organize regular communications with colleagues of the teaching hospitals, involve student representatives in the process by having them promote the study among their classes, and regularly have the study leaflet as the desktop image of computers in the relevant hospital departments. In addition, students who refer a patient for inclusion in the protocol receive extra points for their periodontology practical examination and the patient is referred back to them for the next phases of periodontal therapy.

### Randomization

Patients are randomized using a computerized and centralized system via a specific website. Patients are one-stage randomized, stratified on the following potential confounders: investigation center (Lyon versus Liège), patient gender (male versus female), and patient current tobacco status (currently a tobacco user versus not currently a tobacco user). Because all patient characteristics must be entered in the e-CRF before interventions are assigned, sequence concealment is secured.

### Implementation

The stratification algorithm was implemented by statisticians and methodologists independently of investigators. Patients are assigned to intervention arms by the center coordinator.

### Intervention

#### Visit 1

An investigator verifies the eligibility criteria at the first visit (T0). The patient is informed about the study both orally and by means of an information letter. Patient consent is sought and registered according to the national regulations. The patient’s inclusion is then formalized by the creation of a patient file in the e-CRF.

A clinical examination is performed to assess periodontal health and level of oral hygiene. Other oral conditions (such as dental caries, temporomandibular joint (TMJ) disorders, failing restorations, etc.) will be further treated by referrals inside the dental teaching hospital according to usual procedures and will not be considered in this study. Whenever clinically and ethically possible, the treatment of such conditions will be delayed for a maximum of 8 weeks until the end of the study in order not to interfere with the evaluation of periodontal parameters (e.g. reducing local plaque retention factors by repairing or replacing a failing filling). If treatment cannot be delayed, it will be recorded in the e-CRF.

Then, a 20-min MI session is conducted. All investigators apply the same structure of MI to all patients following a dedicated protocol available in the study folder. This MI protocol is arranged around five themes: reason for seeking consultation, dental and medical history, everyday consequences of periodontal or dental problems, current routine of oral hygiene, and knowledge of and willingness to obtain required dental and medical care. Such a framework was previously validated in a RCT and led to better results than patient instruction alone [36]. The patient is instructed and motivated for a tailored regimen of oral hygiene following international recommendations: twice daily electric toothbrushing with a toothpaste containing fluoride agents, the use of dental floss or interdental brushes, and adjunctive chemical plaque control agents if needed [11–13, 41–43]. Adjunctive chemical plaque control agents could be used for instance in the case of intense gingivitis, to help reduce the total bacterial load. Their use would be limited to as few patients as possible and for the shortest duration achievable [41], to reduce interference with results. If such agents must be used, it will be recorded in the e-CRF.

The use of an electric toothbrush, interdental brushes, dental floss, and toothpaste is demonstrated to the patients by investigators. The patient then has to demonstrate the use of these themselves and reinstruction is performed if needed. This MI session is provided to all patients, irrespective of their allocation (test versus control). All the required materials, including the electric toothbrush, are given to the patient during the MI session in sufficient quantity for the study duration. Patients are specifically instructed to use these tools, and only these tools, during the study. An electric toothbrush was chosen as a moderate level of evidence shows that they to lead to better plaque control and better gingivitis reduction than manual toothbrushes [43]. After the study, patients can keep all materials to continue using them. Replacement heads for the electric toothbrush, and refills for interdental brushes, floss, or toothpaste are readily available to the patients and at a relatively low cost. The end of the study should not prevent them from following the same oral hygiene routine in the long run if they so wish.

After the MI session, an outcome examiner conducts clinical data collection. The clinical data collection for the primary outcome is conducted using a dental plaque disclosing agent, with lip and OptraGate cheek retractors (Ivoclar Vivadent France, Saint-Jorioz, France), and under constant suction.

According to the manufacturer’s information, the TriPlaque disclosing agent (GC Europe, Leuven, Belgium) appears as different colors according to biofilm maturation; new plaque appears pink-red, mature plaque (present for > 48 h) blue-purple, extra high-risk plaque (pH < 4.5) appears light blue.

For data collection, the PCR index [44] is used with six sites per teeth (disto-buccal, buccal, mesio-buccal, mesio-lingual, lingual, disto-lingual). All colors are considered to indicate the presence of plaque and therefore the site is coded “1” by the outcome examiner. If no plaque is present, the site is coded “0”. The examination is conducted visually using loupes and a loupe-mounted light-emitting diode; during the examination a periodontal probe is used on the tooth surface with an aim to avoid false positives and false negatives.

The clinical examination for the secondary outcome is conducted immediately thereafter. A periodontal probe is used to assess the BOP index with six sites per tooth (disto-buccal, buccal, mesio-buccal, mesio-lingual, lingual, and disto-lingual). Criteria for the site to be coded “1” is bleeding within 30 s of probing [45], otherwise the site is coded “0”.

Then, the investigator records the personalized oral hygiene video. This video is recorded for all patients. The investigator uses a loupe-mounted miniature camera connected to a computer (Futudent Educam, Novocam Medical Innovations Oy, Helsinki, Finland). The video lasts approximately 3 min and displays the use of the electric toothbrush, the interdental brushes, and the dental floss. It contains audio instructions summarizing what was said to the patient during the consultation, and is to be watched while brushing the teeth.

After the video recording, the clinical part of the consultation is finished. Patients are then randomized by the center coordinator. Patients allocated to the control group are not given any further detail beyond their allocation. Patients allocated to the test group are registered in the text messages system and their personalized video is uploaded to the cloud portal by the investigator. The investigator instructs them on how the cloud portal and the text messages system work. Both the cloud portal and the text messages system are secured (see “Data management”, “Ethical considerations”, and “Discussion”). The cloud portable is accessible via any smartphone, tablet or computer with a web browser, irrespective of their operating system, and there is no need to install an application in an attempt to maximize technical compatibility. As no application is installed, no notifications will be used to remind the patient about watching the video: only text messages will be used.

The difference between control and test groups is the availability of the personalized oral hygiene video through the cloud server and the registration of the patient in the interactive text messages system. The two follow-up appointments are scheduled, and the visit is brought to an end. Patients in either of the two groups are not contacted by investigators between visits, except for administrative reasons such as rescheduling an appointment.

#### Text reminders

All text reminders sent to patients have the same structure: salutation, a piece of information on oral hygiene and oral health, a question as to whether the patient is compliant to the oral hygiene regiment that was prescribed, an invitation to view the oral hygiene video and a direct link to it, a recall of the date of the next appointment, greetings, and a link to opt out of the text reminder service (as required by law). The information on oral hygiene and oral health varies across messages, in an attempt to keep patients interested in the messages by avoiding repetition and providing them with new information. This piece of information is chosen in a randomized fashion, and once it has been used, it cannot be used in further messages.

Twelve messages are automatically sent during over the course of the study based on the following schedule: the day of visit 1 (V1), the day after V1, 2 days after V1, a week after V1, 2 weeks after V1, 3 weeks after V1, the day before visit 2 (V2), the day of V2, 1 week after V2, 2 weeks after V2, 3 weeks after V2, and 1 day before visit 3.

The patient can answer the text messages to tell the investigators if he is or is not adherent with the oral hygiene routine that was prescribed to him. To do that, he can reply to the text message he received. The system will register his answer in the database and initiate an automated reply. If the patient is adherent, the reply will include elements of positive reinforcement. If the patient is not adherent, the reply will attempt to play down the situation, reassure the patient and offer to discuss this issue at the next appointment. Enough messages have been written to ensure that they are never repeated. All interactions with the patient through text messages are registered in a database to allow descriptive analysis.

#### Visit 2

At the second visit (T0 + 4 weeks), patients are first asked to complete a self-administered electronic questionnaire about their motivation and behavior in terms of oral health [46] that is directly linked to the e-CRF and investigators are blinded to answers. Then, a short (< 5 min) recall about oral hygiene methods is proposed to the patient and conducted by the investigator if needed.

A brief clinical examination is conducted, and the outcome examiner then collects the clinical data. Data collection is conducted following the same protocol as for the first visit. Results are delivered to both the investigator and the patient, and are stored in both the e-CRF and the patient’s electronic healthcare records.

The investigator then conducts full-mouth supra-gingival and juxta-gingival tooth scaling and polishing (Acteon ultrasonic tip number 1 and Newtron LED handpiece, Satelec Acteon, Merignac, France). The investigator ensures thorough removal of calculus but avoids instrumenting the gingival pockets. Following completion of the scaling, patients are invited to rinse with a solution containing 0.12% chlorhexidine digluconate (Paroex, Sunstar France, Levallois Perret, France). Additional supplies of interdental brushes and floss are given to the patient if required. The next appointment is confirmed and the second visit is brought to an end.

#### Visit 3

At the third visit (T0 + 8 weeks), patients are first asked to complete the same questionnaire about their motivation for and behaviors in oral health they completed at the second visit. Then, a short (< 5 min) recall about oral hygiene methods is proposed to the patient and conducted by the investigator if needed.

A brief clinical examination is conducted, and the outcome examiner then collects the clinical data following the same protocol as for the first visit. Results are delivered to both the investigator and the patient, and are stored in both the e-CRF and the patient’s electronic health records.

At the end of the appointment, the patient is asked to complete a satisfaction questionnaire specially designed for dental care (Dental Visit Satisfaction Scale [47]). Again, data are directly entered by the patient into the e-CRF and investigators are blinded to the answers.

Participation of the patient in the study is complete at the end of the third visit. Should the patient require further treatment, they are referred to the relevant departments following standard hospital protocols. If the patient does not need further treatment, they are informed that they should seek an appointment in 1 year for a check-up, either with the staff of the dental teaching hospital or with another dental care provider. The possibility to consult with another dental care provider is by law an inalienable right of the patient. The Ethics Committee emphasized that the patient should be reminded of this.

Various strategies are implemented to improve adherence to intervention protocols: the investigators follow dedicated written protocols to structure the MI (T0), to harmonize the oral hygiene videos (T0), and for recalls about oral hygiene (T0 + 4 weeks and T0 + 8 weeks).

### Outcome measures

Data collection is performed by three blinded, independent, calibrated outcome examiners at 0, 4, and 8 weeks after patient inclusion. For the primary objective (oral hygiene) and the main secondary objective (gingival inflammation) the outcome will be the value of indices collected during the third visit (8 weeks of follow up). Values range from 0 to 100%.

Two additional secondary objectives will be also considered: patient satisfaction after 8 weeks, and patient motivation for change after 4 and 8 weeks in each treatment arm. For the first secondary objective (patient satisfaction after 8 weeks), the Dental Visit Satisfaction Scale questionnaire [47] comprises 10 multiple choice questions (MCQ) scored on a 5-point Likert scale, and explores three aspects of patient satisfaction: information-communication, understanding-acceptance, and technical competence (Additional file 1). For the second secondary objective (patient motivation for change after 4 and 8 weeks), a questionnaire featuring 12 questions (MCQ or binary) derived from previous publications is used [46] (Additional file 2). Results from this questionnaire would allow a preliminary understanding of psychological factors predictive of patient motivation and readiness to change behavior.

### Data collection methods

To promote data quality, investigators and outcome examiners, who will assign scores, are trained in the plaque and gingival inflammation indices by means of group training sessions in real-life patients. To evaluate calibration on the primary outcome (plaque control), investigators and examiners are trained using a large number of pictures of teeth; inter-rater agreement is measured using the Kappa coefficient. Group training sessions and inter-rater agreement assessment are conducted before the beginning of patient inclusion.

For the primary and the main secondary outcome measures, data collection forms are generated through a public online platform [48]. For the patient-reported outcomes (remaining secondary objectives), patients enter their answers to the questionnaires directly on a dedicated and password-protected page of the e-CRF. No particular reward to promote participant retention and complete follow up has been instated.

### Data management

Investigators and outcome examiners enter the data into the e-CRF. Fields cannot be left blank. Interactive data controls will be applied for value ranges and presence of impossible values and for between-form coherence. A patient-operated e-CRF allows for the collection of patient-reported outcomes without the intervention of the study investigators. Data will be kept anonymous, with high-level security storage, and encryption of all data transfers, in compliance with French regulatory and European Clinical Research Infrastructure Network (ECRIN) requirements [49].

### Statistical methods

The statistical unit for analysis will be the patient. All of the patient’s teeth are considered for data collection but unerupted, impacted, or fractured teeth are excluded (site-related exclusion criteria). Six sites per tooth are examined.

The demographic and clinical characteristics of patients will be described for both the test and control groups with mean and standard deviation or median and interquartile ranges for quantitative variables, and number of subjects and percentages for qualitative variables. The analyses will be performed according to the intention-to-treat principle, modified to include only patients with available outcomes.

To respond to the primary objective, the mean plaque score in the test and control groups at 8 weeks will be calculated and compared using Student’s *t* test for unpaired data. The plaque score at the third visit will be modeled using a hierarchical model accounting for the correlation of the measures per patient and any possible inter-practitioner heterogeneity. The model will be adjusted on randomization stratification factors (center of inclusion, patient gender, and tobacco use) as well as the baseline value collected at the first visit. If necessary, other adjustment factors might also be considered. The main secondary objective (bleeding on probing) will be analyzed using Student’s *t* test (nonparametric test whenever relevant).

In cases of patient non-compliance with follow-up visits, sensitivity will be analyzed using the last outcome examiner’s assessment (last observation carried forward). Additionally sensitivity will be analyzed considering all missing data as equal to the test or control group’s mean values for the given visit. Candidate factors for subgroup analyses are patient age, patient gender, diabetic status, tobacco use, and at least one general medical condition.

### Data monitoring, harms, and auditing

The data will be monitored by an independent clinical research assistant who will compare the data entered in the e-CRF with those in the patient’s electronic health records. In the case of disagreement, the patient’s physician will be asked to clarify the data. No interim analysis is planned. Concerning harms monitoring, specific adverse event forms can be accessed in the e-CRF. Trial management may be audited by the Agence Nationale pour la Sécurité du Médicament et des Produits de Santé (ANSM, French medicines agency) at any time; the audit would be independent of investigators and the sponsor.

### Dissemination of results

The Consolidated Standards of Reporting Trials (CONSORT [50]) guidelines, CONSORT Extension for Patient-Reported Outcomes in Randomized Trials (CONSORT PRO extension [51]) and ﻿the CONSORT Extension for Electronic and Mobile Health Applications and online Telehealth (CONSORT-EHEALTH [52]) guidelines will be used to report the results of this study and the results will be published in international peer-reviewed journals. Authors of the publications will be those involved in the elaboration of the protocol, the implementation and conduct of the trial, and the drafting of the manuscript and report. The results related to the main objective will be authored by the coordinator, the methodologists, the investigators, the outcome examiners, and others who will have significantly contributed to the planning of the trial, its implementation, or the drafting of the report.

A summary of the study results will be posted on ClinicalTrials.gov to allow general access to the findings. Study participants will be informed about the study results.

## Discussion

The inclusion and non-inclusion criteria for this RCT require patients to have at least 20 teeth and 4 premolar-molar interdental sites. Such criteria can lead to the exclusion of patients affected by severe periodontal disease with several lost teeth. The external validity of the study for this particular patient population should be interpreted with caution. Yet, two reasons led to this choice. First, the eHealth concept proposed in this trial is applicable in primary prevention (prior to the onset of any periodontal disease), as well as in secondary prevention (gingivitis) and tertiary prevention (periodontitis). Second, the statistical unit for analysis is the patient. As such, estimates for patients with few remaining teeth could be less reliable and the planned statistical analysis does not allow for weighting results from different patients according to number of teeth. Other inclusion and non-inclusion criteria and the overall study methodology are in line with the recommendations from the Cochrane Oral Health Group [38, 53].

Restricting patients’ age to a younger group could be questioned, as engagement of patients with eHealth is negatively correlated with age although correlation coefficients are small [54, 55]. But other factors such as educational attainment level [54, 55] and technophilia [56] also influence patients’ attitudes towards eHealth. As such, researchers and developers should take into consideration these factors and others in order to overcome barriers to eHealth in the public at whom they are aimed [57, 58]. Application design and user experience are important elements in that regard, especially for an elderly population [59]. By keeping the cloud interface as minimal as possible, we aimed to make it as accessible as possible. Also, this influenced the choice for a platform that does not require to download and install an application, as it runs entirely within the web browser. Finally, the inclusion of a direct link to the patient’s video in the text reminders further reduces such obstacles, as patients only need to click on that link and enter their credentials to consult their video.

The choice of a given plaque index has an impact on the statistical analyses - and likely on the outcomes. Several plaque indices have been developed, with different fields of application [60]. The modified PCR index chosen for this study records presence or absence of plaque (binary outcome) on six sites per tooth. Some indices are more research-oriented such as the Turesky modification of the Quigley-Hein Plaque Index that records plaque as an ordinal outcome with six levels [61, 62] or the Rustogi/Navy Plaque Index that records plaque still as a binary outcome but on nine sites per tooth [63]. An ordinal outcome and more sites per tooth dramatically increase the sensitivity of the index, thus allowing the detection of smaller differences between groups [64, 65]. While the original PCR index proposed by O’Leary et al. used four sites per tooth [44], it was decided for this RCT to use six sites per tooth to achieve a balance between clinical practicability and precision for plaque assessment. Also, using a plaque disclosing agent facilitates the detection of plaque [66] but a number of false positives were detected during the calibration phase of the trial. Taking this finding into consideration, outcome examiners were instructed to use a periodontal probe on the tooth surface when in doubt, to clear any uncertainty about the presence or absence of plaque.

In terms of internal validity, the sources of bias are limited by the use of centralized randomization (selection bias), strict prospective data record and monitoring (information bias), and blinded outcome examiners (performance and detection bias). However, because of the nature of the investigation, the patients cannot be blinded. Also, investigators are not blinded but as patients are randomized at the end of the first visit, it ensures that investigators are not biased when performing the MI and recording the video.

A factorial design would have allowed us to explore any interaction between the patient education strategy (control versus test eHealth strategy) and the type of toothbrush recommended during the consultation (electric versus. manual toothbrush). One might consider that correct demonstration is of major importance for manual toothbrushes and that electric toothbrushes are less dependent on a patient’s brushing technique. Two reasons led to the choice of a two parallel-arm design with electric toothbrushes for all participants. First, the sample size for a full factorial design (including interactions) would have been of 508 patients without taking into account patients lost to follow up. This was beyond the recruitment capacity of the centers involved so either electric or manual toothbrushes had to be selected for all patients. Second, a Cochrane review established with a moderate level of evidence that electric toothbrushes are more effective than manual toothbrushes for plaque removal by 11% and up to 21% when follow up is over 3 months [43] so the decision was made to favor electric toothbrushes.

To use only one type of toothbrush for all patients also helps to suppress potential bias due to the variation in cleaning efficacy between different models of toothbrushes, manual or electric. However, to generalize the results to all toothbrush designs and validate the added value of our eHealth solution (video + SMS), further studies would be needed.

While electric toothbrushes are more expensive than manual ones, cost to patients was not an issue for this trial as all oral hygiene materials are provided to them. Lastly, some electric toothbrushes can be connected to smartphones, which sounds a promising area of research for enhanced interaction with the patients and adherence monitoring.

Patient compliance for oral hygiene is of paramount importance for oral health [15, 67], and patient motivation is the first step in periodontal therapy and instrumental care should not be initiated before plaque control is satisfactory. The focus of the present study is to evaluate the added value of a eHealth platform (video + SMS) for the acquisition of an effective oral hygiene routine. As such, an 8-week follow up is acceptable, and fits with the recommendations from the Cochrane Oral Health Group [38, 53].

However, when patients enter supportive periodontal therapy, oral hygiene should be maintained over time in order to entail long-term tooth loss: in a 10-year cohort study, Eickholz et al. found that an increase of 10 points in the PCR index was associated with a risk ratio of 1.57 for tooth loss [16]. As such, should the stability over time for oral hygiene adherence be investigated, a much longer follow up, for instance 1 year, would be required. A 1-year follow-up visit could be organized after further approval by the Ethics Committee. Such a possibility has been expressly incorporated in the file submitted to the Ethics Committee. If a 1-year visit is scheduled, the calibrated researchers from the original 8-week study will be responsible for it, in order to standardize the protocol.

The eHealth concept assessed in this RCT aims to take advantage of MI for patient motivation and to create a supportive environment at home through the use of videos and text messages. According to the 2016 Eurostat survey, respectively 78% of Belgian and 71% of French residents used their smartphones to access the Internet as often or more often than desktop computers, laptops, and tablets [68]. This motivated the choice of a cloud platform compatible with smartphones so that patients can view their videos any time.

As the video is recorded by a healthcare professional, the information delivered through it is of adequate quality. The burden of recording the video is minimized by the small size of the camera, as it can be kept on the dental loupes all the time. Also, the structure of the consultation can be optimized by each practitioner according to their wishes. Recording the video requires less than 5 min additionally for the clinician, which is reasonable as the latest findings suggest that 50% of European periodontal practitioners spend more than 15 min for the first phase of patient education before instrumental care [69].

Overall, several barriers and facilitators have been identified for the implementation of eHealth solutions [70]. A recent systematic review indicates that one of the key obstacles is when systems require users to manually enter large amounts of data [71]. With the computer framework used in this RCT, while recording and sharing the video is straightforward, the system supporting text messages is more cumbersome. A gateway to the telephone network had to be developed separately from the electronic health records system of the teaching hospital and of the camera software. When registering a new patient in the text messages database, several steps are needed, and the process takes 5–10 min. While acceptable in a research environment, these extra steps might affect productivity in a private dental practice. Further integration of such systems into various practice management software could help streamline the process.

The legislative and regulatory environment should also be taken into consideration. For the video recording, it might be necessary to seek and record patient consent in some settings, as it would be for photographs. Regarding the cloud platform, the solution proposed by the camera manufacturer fully complies with the new European Union General Data Protection Regulation requirements and relies on a certified cloud provider (Microsoft Azure). Similarly, the telephone network gateway is secured, and all communications are encrypted. If this eHealth concept is to be disseminated to private practices, practice owners and service providers should pay great attention to the applicable legal and regulatory requirements, in particular to who is responsible for data confidentiality.

The present research focuses on the assessment of a novel eHealth concept linking proven behavior change techniques and mobile technology. After motivation for oral hygiene in the dental office, the proposed concept aims to help keep the link with the patient at home and strengthen their long-term commitment for a good oral hygiene routine. The availability of new data should help make a case for an increased use of eHealth solutions by dental practitioners.

## Trial status

The trial is currently in the recruitment phase.

## Supplementary information

**Additional file 1.** Dental Visit Satisfaction Scale questionnaire.

**Additional file 2.** Patient-declared behavior change questionnaire.

## Data Availability

Access to the full protocol can be granted to anyone upon request. The datasets generated and/or analyzed during the current study are available from the corresponding author on reasonable request.

## Abbreviations

ANSM: Agence Nationale pour la Sécurité du Médicament et des Produits de Santé (French medicines agency, independent state agency)
BOP: Bleeding on probing
CNIL: Commission Nationale de l’Informatique et des Libertés (National Commission for Information Technology and Individual Freedom, independent state agency)
CONSORT: Consolidated Standards of Reporting Trials
CONSORT-EHEALTH: Consolidated Standards of Reporting Trials of Electronic and Mobile HEalth Applications and onLine TeleHealth
CONSORT-PRO: Consolidated Standards of Reporting Trials - Patient-Reported Outcomes
e-CRF: Electronic case report form
ECRIN: European Clinical Research Infrastructure Network
MCQ: Multiple choice question
MI: Motivational interviewing
RCT: Randomized controlled trial
SPIRIT: Standard Protocol Items: Recommendations for Interventional Trials
SPIRIT-PRO: Standard Protocol Items: Recommendations for Interventional Trials - Patient-Reported Outcomes
