# Supplementary material for: GoPerio - impact of a personalized video and an automated two-way text-messaging system in oral hygiene motivation: study protocol for a randomized controlled trial
Source: Trials. 2019 Dec 10;20:699. doi: 10.1186/s13063-019-3738-0 (PMC6905095; doi:10.1186/s13063-019-3738-0)
Supplement: Supplementary file 1 — Additional file 1. Dental Visit Satisfaction Scale questionnaire. [file 13063_2019_3738_MOESM1_ESM.pdf]

Patient satisfaction questionnaire
Visit Three

Assessed at the end of the third visit , just before ending the patient's participation to the story

Answered directly by the patient on-site on **the eCRF**

Investigators and outcome examiners are blinded from the results

Using the **Dental Visit Satisfaction Scale (Corah et al. 1984**) with a **5-points Likert scale**

The Dental Visit Satisfaction Scale Items

On this 5 points-scale, how much do you agree with the following ?

Strongly disagree	Disagree	Neither agree nor disagree	Agree	Strongly agree

Information-Communication

1. After talking with the dentist, I know what the condition of my mouth is.

2. After talking with the dentist, I have a good idea of what changes to expect in my dental health in the next few months.

3. The dentist told me all I wanted to know about my dental problem(s).

Understanding-Acceptance

4. I really felt understood by my dentist.

5. I felt that this dentist really knew how upset I was about the possibility of pain.

6. I felt this dentist accepted me as a person.

Technical Competence

7. The dentist was thorough in doing the procedure.

8. The dentist was too rough when he worked on me.*

9. I was satisfied with what the dentist did.

10. The dentist seemed to know what he was doing during my visit.

**Scored on the opposite direction because of the negative content.*
