# Supplementary material for: GoPerio - impact of a personalized video and an automated two-way text-messaging system in oral hygiene motivation: study protocol for a randomized controlled trial
Source: Trials. 2019 Dec 10;20:699. doi: 10.1186/s13063-019-3738-0 (PMC6905095; doi:10.1186/s13063-019-3738-0)
Supplement: Supplementary file 2 — Additional file 2. Patient-declared behavior change questionnaire. [file 13063_2019_3738_MOESM2_ESM.pdf]

Patient-declared behavior change questionnaire
Visit Two, Visit Three

Assessed a**t the beginning of the second and third visits**

Answered directly by the patient on-site on **the eCRF**

Investigators and outcome examiners are blinded from the results

Using a modified version of a **dedicated questionnaire developed by Saito et al. 2009**

**Frequency of toothbrushing (per day)**				
Less than once a day	Once a day	Twice a day	Three times a day	More than three times a day

**Use of toothpaste**	
No	Yes

**Approximal cleaning (dental floss or interdental brushing)**	
No	Yes

**Use of mouthwash or other products**	
No	Yes

**How often do you check your teeth or mouth in a mirror?**			
Almost never	A few times/month	A few times/week	Everyday

**How would you rate your desire to keep your teeth?**				
Very weak	Weak	Fair	Strong	Very strong

**How much are you willing to do to improve your oral health?**		
Not very much	I am willing to take some action	Anything necessary

**Have you been maintaining regular dental check-ups?**	
No	Yes

**How much do you follow your dentist's or dental hygienist's advice on oral hygiene care?**			
Never	Not very much	Sometimes	Always

**How important is the prevention of cavities or gum diseases for you?**		
Not at all important	Somewhat important	Very important

**Are you willing to take on new challenges and/or change your daily routine?**	
No	Yes

**To what degree do you feel that the actions you take have an impact on your own oral health?**		
I cannot do much about it	Not sure either way	My actions play a significant role
